# Comparison of time to therapy initiation, therapeutic strategies and survival in patients with MDS IB1, IB2 and AML-MR using data of the duesseldorf registries

**DOI:** 10.1007/s00277-026-07250-3

**Published:** 2026-08-26

**Authors:** Felicitas Schulz, Catharina Mundt, Annika Kasprzak, Paul Jaeger, Andrea Kuendgen, Corinna Strupp, Norbert Gattermann, Kathrin Nachtkamp, Sascha Dietrich, Guido Kobbe, Ulrich Germing

**Affiliations:** Department of Hematology, Oncology and Clinical Immunology, Heinrich-Heine-University Düsseldorf, Moorenstr. 5, Düsseldorf, 40225 Germany

**Keywords:** Myelodysplastic syndromes, MDS, Acute myeloid leukemia, AML-MR

## Abstract

In 2022, the ICC redefined AML by lowering marrow blast count to 10% herewith integrating former MDS IB2 into AML. To evaluate the clinical implications of this reclassification, we analyzed data of our Duesseldorf registries regarding different aspects in patients with MDS IB1, IB2 or AML-MR and included 928 patients suffering from IB1 (213 patients), IB2 (279 patients) and AML-MR (436 patients). We analyzed time to initiation of treatment as well as treatment strategies and survival times. Time to initiation of therapy was significantly shorter in patients with AML-MR compared to MDS IB2 and IB1 (0.5 vs. 2 vs. 5 months, *p* < 0.001). Median overall survival was 12 months in patients with AML-MR, 19 months in MDS IB2 and 27 months in MDS IB1 (*p* < 0.001). Regarding most intensive treatment, median overall survival did not differ significantly in patients undergoing allografting or induction chemotherapy but was significantly longer (*p* < 0.001) in patients with MDS IB1 or IB2 compared to AML-MR receiving HMA (29 vs. 20 vs. 9 months), low dose chemotherapy (18 vs. 11 vs. 1 months) or BSC only (16 vs. 9 vs. 2 months). The significantly longer time to treatment initiation together with the prolonged median overall survival and survival following treatment with hypomethylating agents (HMAs) or low-dose chemotherapy suggests biologically relevant differences between AML-MR and MDS-IB2 that may not support their classification as a single disease entity.

## Introduction

 Myelodysplastic syndromes (MDS) comprise clonal disorders of the hematopoietic stem cell characterized by dysplasia of the bone marrow and the increased risk of transforming into acute myeloid leukemia (AML) [1]. With a median age of 75 at time of diagnosis, patients suffer from different degrees of hematopoietic insufficiency with pancytopenia as worst-case scenario resulting in a frequent need of transfusion therapy and a permanent risk of infectious complications. Therefore, prognosis and treatment options differ widely between patients, making precise prognostication even more important [2]. Acute myeloid leukemia (AML) is a heterogeneous disease of older patients with a median age of 72 years at initial diagnosis [3]. Treatment-related mortality as well as therapy resistance confer a poor prognosis [4, 5]. Since 2022, there are two competing classifications of myeloid neoplasms and acute leukemias, the updated 5th edition of the WHO as well as the ICC, both based on cytomorphological, cytogenetical and molecular characteristics [6, 7]. They differentiate between ‘MDS with defining genetic abnormalities’ and ‘MDS, morphologically defined’ with three subcategories each [6, 7]. Paraphrased, the two classification systems differ in their underlying priorities, with the WHO classification (through the additional consideration of histology) placing even a stronger emphasis on morphological features than the ICC [8]. One of the most significant differences probably was that the WHO classification still defined AML presenting with a minimum of 20% myeloid blasts in the bone marrow (in the absence of AML-defining genetic lesions) while the ICC constituted an AML diagnosis starting from 10% myeloid marrow blasts [6, 7]. As hematologic neoplasms like myelodysplastic syndromes and acute myeloid leukemia represent complex diseases by nature, competing classifications for the same disease add avoidable complexity for patients, treating physicians as well as pathologists and interfere with both treatment decisions as well as prognostication and enrollment of patients in clinical trials [9]. Therefore, the simultaneous publication of the WHO and the ICC classification in 2022 roiled the world of myeloid neoplasms and made physicians ask for a re-unified classification abolishing the main difference regarding the blast cut-off to call patients’ disease either myelodysplastic syndrome or acute myeloid leukemia [10]. During the last four years, there were different proposals to merge WHO and ICC [11]. In 2024, Nachtkamp et al. also proposed a validation-based merged classification using data of our Düsseldorf MDS Registry and postulated that the WHO 2022 classification offered clearer definitions and addressed underestimated features of MDS by including histopathology [12]. Their proposal based on our Registry data was to use morphologically and genetically defined MDS subgroups, but to keep the well-known blast cut-off < 20% to distinguish between IB2 and AML (the exact number of blasts was only overruled in case of biallelic TP53-mutation due to its bad prognosis comparable to AML patients with this genetic alteration) [12]. With regard to a planned meeting of MDS experts in 2026 and the aim to update the classification of MDS, we analyzed data of almost 1000 patients with MDS IB1, IB2 and AML-MR of our Düsseldorf MDS and AML Registries regarding disease biology, treatment strategies and survival to validate or contradict the blast cut-off alteration of the ICC.

## Methods

In this retrospective study, we analyzed 928 patients from the Düsseldorf MDS registries who were diagnosed and treated at the Department of Hematology of the University Hospital Düsseldorf between 1969 and 2023. Although the registries comprise nearly 11,000 patients, only 928 fulfilled the inclusion criteria (IB1, IB2, and AML-MR) for the present analysis as eligibility required not only detailed information on treatment exposure but also complete documentation of the exact initiation date of each therapeutic intervention. Patients were prospectively enrolled in the registries by their treating physician at the time of diagnosis and underwent regular follow-up according to institutional practice. Patients had a median age at diagnosis of 66 years (60% male) and suffered from IB1 (213 patients), IB2 (279 patients) and AML-MR (436 patients). Diagnoses were assigned retrospectively according to the WHO 2022 classification based on the peripheral blood or bone marrow blast count assessed by our morphologic laboratory. Allografting was considered as the most intensive treatment option followed by induction chemotherapy while best supportive care was the least intensive. Treatment with hypomethylating followed induction chemotherapy regarding hierarchy of treatment intensity while low-dose chemotherapy was considered as less intensive. Molecular analyses were performed using next generation sequencing. Descriptive statistical analyses were performed using the Statistical Package for the Social Sciences (SPSS) version 30 (SPSS, Chicago, IL, USA). Clinical and hematologic characteristics at the time of diagnosis were compared between WHO classification groups. Categorical variables were analyzed using the c² test. Normally distributed continuous variables were compared using one-way analysis of variance (ANOVA) or Welch’s ANOVA when the assumption of homogeneity of variances was violated. Non-normally distributed continuous variables were compared using the Kruskal–Wallis test. Multivariable analyses were performed using Cox proportional hazard models. A two-sided p-value of less than 0.05 was considered as statistically significant. The probability of survival was estimated using Kaplan–Meier method [13]. Group comparisons of Kaplan-Meier curves were performed using log-rank test. The study was conducted in accordance with the Declaration of Helsinki and approved by the Ethics Committee of the Heinrich-Heine University in Duesseldorf (No. 3973).

## Results

**Fig. 1:**
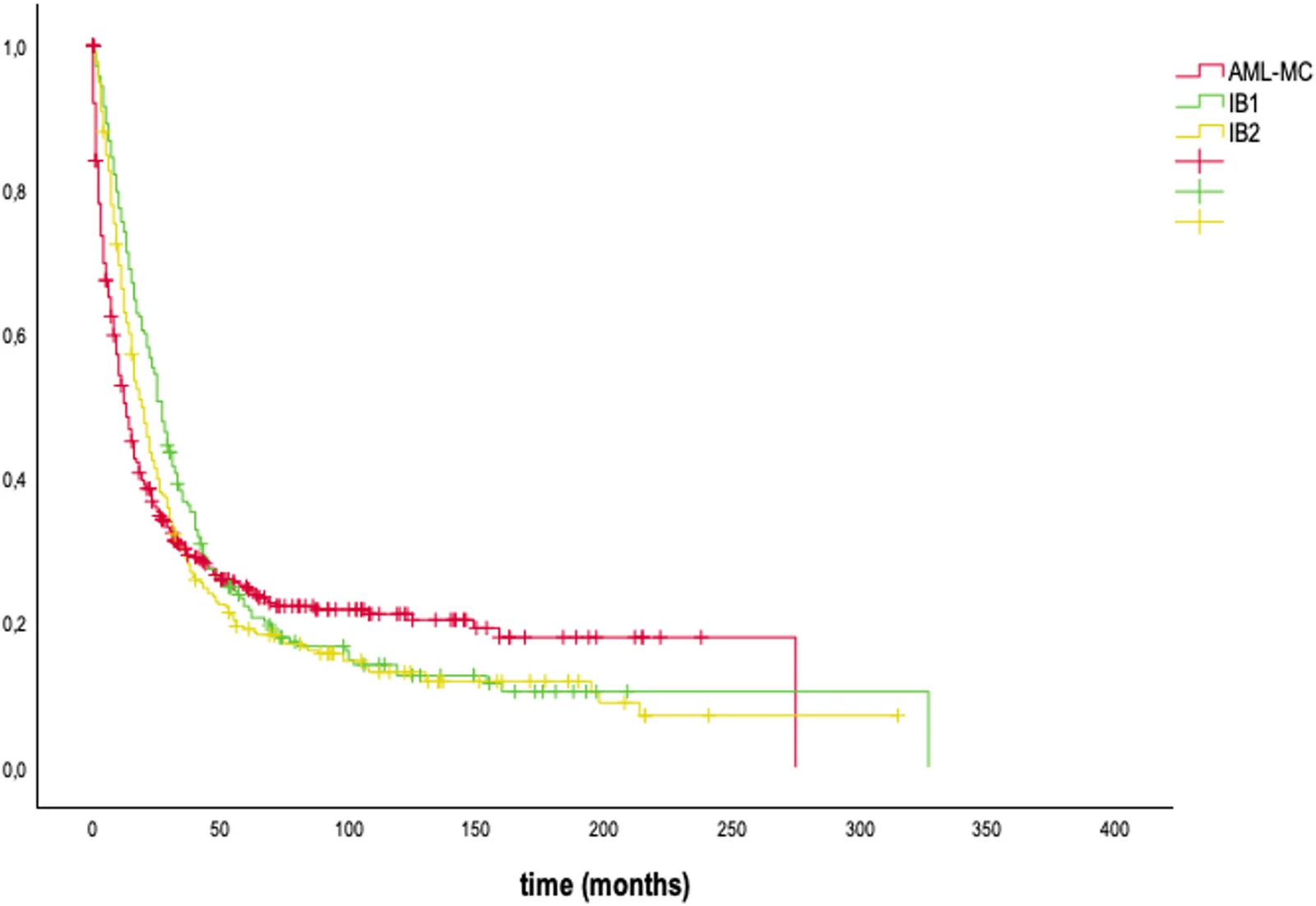
Overall survival of the whole cohort according to treatment and underlying disease

**Fig. 2:**
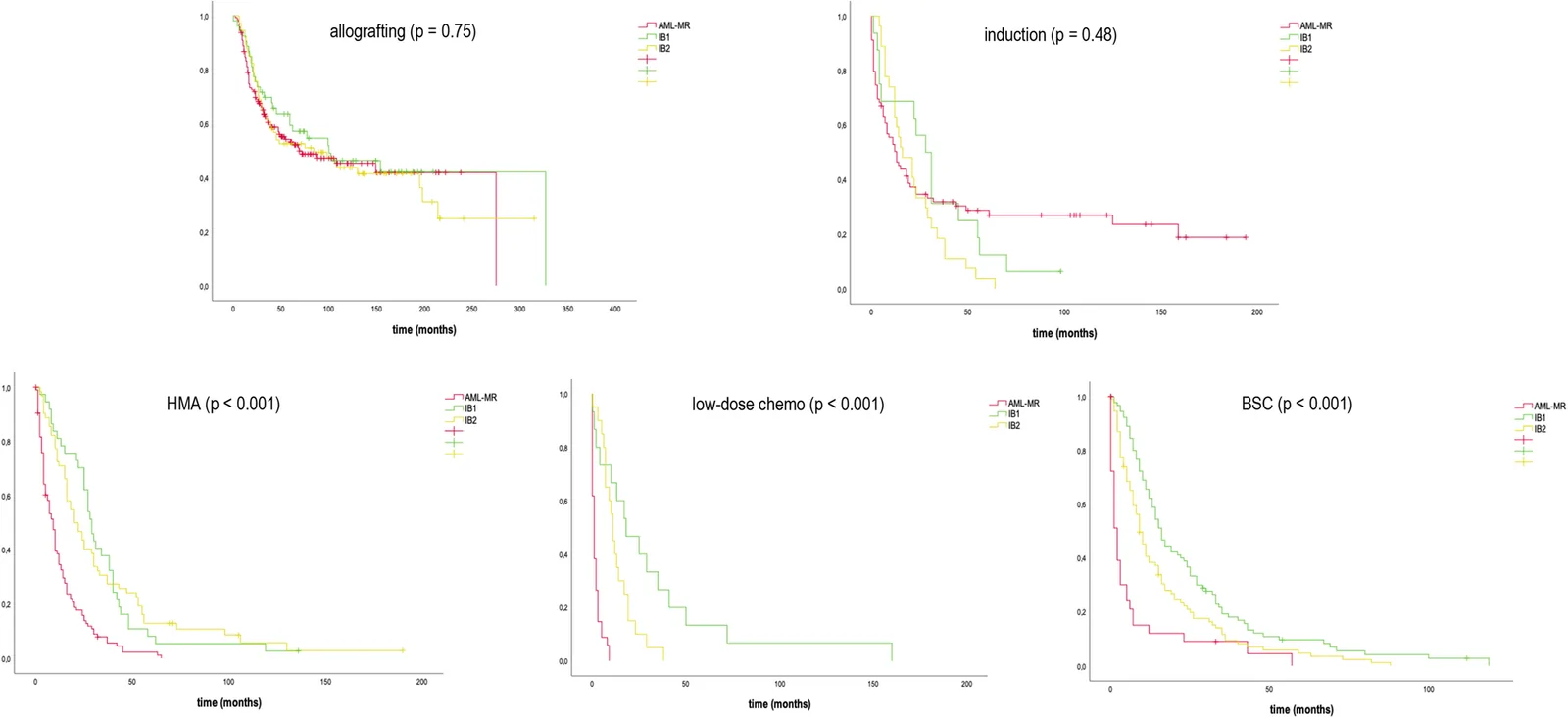
Overall survival of the whole cohort according to the underlying disease

Patient characteristics at the time of diagnosis are shown in Table 1. At the time of our analyses, 169 patients (18%) were still alive (101 of 436 AML-MR patients (23%), 37 of 279 IB2 patients (13%) and 31 of 213 IB1 patients (15%) while a total of 10 patients (1%) were lost to follow-up. Median follow up was 16.5 months (range 1–327) and was 27 months for patients with IB1 (range 1–327), 19 months in patients with IB2 (range 1–315) and 12 months in patients suffering from AML-MR (1–275 months). There were no statistically significant differences regarding gender or age between the three groups. Patients with AML-MR per definition had a statistically significant higher medullary blast count (*p* < 0.001) as well as lower platelets (*p* < 0.001 and 0.002) and higher LDH levels (*p* < 0.001 and 0.001) than both MDS groups. Differences regarding number of white blood cells and hemoglobin levels have only been statistically significantly different regarding MDS IB1 (*p* = 0.008 and 0.01). A statistically significant higher number of AML patients had received prior cytotoxic therapies compared to both cohorts of MDS patients. Overall, 194 of 492 MDS patients (39.4%) experienced transformation to AML. Table 2 shows the most intensive treatments patients received according to their underlying disease. A higher proportion of AML patients underwent intensive therapies like allografting or induction chemotherapy compared to patients with IB1 or IB2 (*p* = 0.04 and < 0.001) while more MDS patients were treated with best supportive care only (*p* < 0.001). Mean time to initiation of therapy was 3 months (range 0 to 67 months) and was significantly shorter in patients with AML-MR compared to MDS IB2 and IB1 (median 0.5 (range 0 to 48) vs. 2 (0 to 42) vs. 5 (0 to 67) months and mean 1 vs. 5 vs. 9 months, *p* < 0.001). Median overall survival in months according to underlying disease and most intensive treatment is shown in Table 3. Median overall survival of the whole cohort was 16.5 months with 12 months in patients with AML-MR, 19 months in MDS IB2 and 27 months in MDS IB1 as shown in Fig. 1. Regarding most intensive treatment, median overall survival did not differ significantly in patients undergoing allografting (*p* = 0.75) or induction chemotherapy (*p* = 0.48) but was significantly longer (*p* < 0.001) in patients with MDS IB1 or IB2 compared to AML-MR receiving HMA (29 vs. 20 vs. 9 months), low dose chemotherapy (18 vs. 11 vs. 1 months) or BSC only (16 vs. 9 vs. 2 months) as shown in Fig. 2. Multivariable analyses were performed to evaluate potential confounders testing variables like patient’s age, gender, year of diagnosis, disease etiology (primary vs. post-cytotoxic therapy) and cytogenetic risk. Variables of statistical significance were WHO category (AML-MR vs. IB1 vs. IB2, *p* < 0.001), most intensive treatment (*p* < 0.001), cytogenetic risk (*p* < 0.001) as well as patient’s age (tested as younger or older than the median age of 66 years, *p* = 0.004). All other variables including the diagnostic era as well as the disease etiology or sex category could be excluded as confounders.

**Table 1:** Patient characteristics at time of diagnosis

	All patients (*n* = 928)	AML-MR (*n* = 436)	IB2 (*n* = 279)	IB1 (*n* = 213)	*p*-value (IB1 vs. IB2, IB2 vs. AML, IB1 vs. AML)
Gender, *n* (%)					
Male	550 (59.3)	247 (56.7)	170 (60.9)	133 (62.4)	0.294
Female	378 (40.7)	189 (43.3)	109 (39.1)	80 (37.6)	
Age, median (range)	66 (19–93)	66 (19–92)	67 (19–92)	65 (19–93)	0.518, 0.420, 0.999
Year of diagnosis, n (%)					
< 1990	83 (8.9)	9 (2.1)	42 (15.1)	32 (15.0)	0.594, < 0.001, < 0.001
1990–2006	161 (17.3)	4 (0.9)	94 (33.7)	63 (29.6)	
2007–2019	560 (60.3)	323 (74.1)	132 (47.3)	105 (49.3)	
≥ 2020	124 (13.4)	100 (22.9)	11 (3.9)	13 (6.1)	
Medullary blast count in %, median (range)	15 (2–99)	39 (2–99)	14 (10–19)	8 (4–9)	< 0.001
Hemoglobin in g/dl, median (range)	9.0 (2.1–15.0)	8.8 (2.1–14.9)	9.3 (4.8–15.0)	9.2 (4.3–15.0)	0.969, 0.01, 0.063
WBC x 1000/µl, median (range)	4.0 (0.2–421)	5.4 (0.2–421)	2.7 (1–61.0)	3.6 (1–64)	0.504, 0.008, 0.054
Platelets x 1000/µl, median (range)	50 (1–962)	45 (3–650)	96 (12–573)	76 (1–962)	0.450, < 0.001, 0.002
LDH U/l, median (range)	316 (94–9034)	343 (94–9034)	223 (165–1630)	234 (155–1492)	0.765, 0.001, < 0.001
Etiology					
Primary, n (%)	691 (74.4)	277 (63.5)	239 (85.7)	175 (82.2)	< 0.001
Post-cytotoxic therapy, n (%)	189 (20.4)	132 (30.3)	31 (11.1)	26 (12.2)	
Incomplete documentation, n (%)	48 (5.2)	27 (6.2)	9 (3.2)	12 (5.6)	
Cytogenetics at time of diagnosis					
Favorable, n (%)	157 (16.9)	8 (1.8)	78 (28.0)	71 (33.3)	< 0.001
Intermediate, n (%)	265 (28.6)	205 (47.0)	40 (14.3)	20 (9.4)	
Adverse, n (%)	270 (29.1)	169 (38.8)	60 (21.5)	41 (19.3)	
Complex karyotype, n (%)	208 (22.4)	131 (30.0)	48 (17.2)	29 (13.6)	
Missing	236 (25.4)	54 (12.4)	101 (36.2)	81 (38.0)	
Molecular analyses at time of diagnosis					
RUNX1, n/patients tested (%)	57/153 (37.3)	39/94 (41.5)	11/32 (34.4)	7/27 (25.9)	
ASXL1, n/patients tested (%)	56/152 (36.8)	33/82 (40.2)	11/34 (32.4)	12/36 (33.3)	
TP53, n/patients tested (%)	46/209 (22.0)	17/79 (21.5)	19/66 (28.8)	10/64 (15.6)	
NPM1, n/patients tested (%)	21/284 (2.3)	21/237 (8.0)	0/25	0/22	
IDH1, n/patients tested (%)	18/183 (9.8)	11/118 (9.3)	6/36 (16.6)	1/29 (3.4)	
IDH2, n/patients tested (%)	18/147 (12.2)	15/119 (12.6)	3/19 (15.8)	0	
SF3B1, n/patients tested (%)	11/57 (19.3)	0/1	4/25 (0.16)	7/31 (22.6)	
FLT3, n/patients tested (%)	2/295 (0.7)	2/256 (0.8)	0/19	0/20	
IPSS-R category					
Very low, n (%)	0	n.a.	0	0	
Low, n (%)	6	n.a.	1 (0.4)	5 (2.3)	
Intermediate, n (%)	75	n.a.	30 (10.8)	45 (21.1)	
High, n (%)	112	n.a.	67 (24.0)	45 (21.1)	
Very high, n (%)	93	n.a.	66 (23.7)	26 (12.2)	
Missing	207 (42.1)	n.a.	115 (41.2)	92 (43.2)

**Table 2:** Most intensive treatment according to underlying disease

	All patients (*n* = 928)	AML-MR (*n* = 436)	IB2 (*n* = 279)	IB1 (*n* = 213)	*p*-value
Allografting, *n* (%)	274 (29.5)	146 (33.5)	75 (26.9)	53 (24.9)	0.04
Induction chemotherapy, n (%)	124 (13.4)	80 (18.3)	28 (10.0)	16 (7.5)	< 0.001
hypomethylating agents (HMA), n (%)	205 (22.1)	106 (24.3)	62 (22.2)	37 (17.4)	n.s.
HMA + BCL2-Inhibition, n (%)	37 (4.0)	33 (7.6)	2 (0.7)	2 (0.9)	n.s.
low-dose chemotherapy, n (%)	70 (7.5)	35 (8.0)	20 (7.2)	15 (7.0)	n.s.
best supportive care, n (%)	218 (23.5)	36 (8.3)	92 (33.0)	90 (42.3)	< 0.001

**Table 3:** Median OS in months according to underlying disease and most intensive treatment

	All patients (*n* = 928)	AML-MR (*n* = 436)	IB2 (*n* = 279)	IB1 (*n* = 213)	*p*-value
Allografting, median (95% CI)	87 (55.7–118.3)	69 (2.3–135.7)	84 (25.5–142.5)	100 (11.2–188.8)	0.75
Induction chemotherapy, median (95% CI)	15 (9.2–20.8)	13 (7.3–18.7)	16 (7.1–24.9)	28 (20.2–35.8)	0.48
hypomethylating agents (HMA), median (95% CI)	15 (12.3–17.7)	9 (6.9–11)	20 (13.1–26.9)	29 (24.8–33.2)	< 0.001
HMA + BCL2-Inhibition, median (95% CI)	8 (5.5–10.5)	8 (5.5–10.5)	---	---	
low-dose chemotherapy, median (95% CI)	4 (1.3–6.7)	1 (0.3–1.7)	11 (8.8–13.2)	18 (2.9–33.1)	< 0.001
best supportive care, median (95% CI)	11 (9.1–12.9)	2 (1–3)	9 (7.0–11)	16 (13.1–18.9)	< 0.001

## Discussion

The question whether myelodysplastic syndromes and acute myeloid leukemia should be conceptualized as distinct entities or as part of a biological continuum remains a central controversy in contemporary hematologic classification. The integration of MDS-IB2 into AML category bears far-reaching implications for both diagnostics and risk stratification as well as prognostication and therapeutic decision-making. On the one hand, it enables earlier therapeutic intervention in patients with high-risk diseases while on the other hand there have always been important concerns regarding the risk of overtreatment and the comparability of clinical trial populations [14]. The optimal boundary between myelodysplastic syndromes and acute myeloid leukemia remains a matter of ongoing debate, especially in the era where molecular and genetic features increasingly challenge morphology-based classification systems [15, 16]. Therefore, we analyzed data of almost 1000 patients of our MDS and AML registries to either support or deflect the new ICC subgroup of MDS/AML-patients. The baseline characteristics of our cohort showed a difference regarding white blood cell count, hemoglobin levels and platelets at diagnosis between patients with IB2 and AML-MR. Patients with IB2 had higher Hb-levels, higher platelets and lower WBC herewith being less at risk for symptoms of anemia or bleeding than patients with AML-MR. According to our data, treating physicians do handle these two disease types differently. There seems to be a difference regarding the perceived urgency of initiating treatment in a timely manner. Reflecting the different disease biology of patients with AML-MR compared to MDS IB2, median overall survival of patients with AML-MR was significantly shorter than in MDS IB2 and IB1 with a difference of 7 months between AML and IB2 and even 15 months between AML and IB1 respectively. The first assumption reading this might be that a higher number of AML patients underwent intensive treatment such as allografting (34 vs. 27 vs. 25%) with higher risk of treatment-related mortality, but looking at median overall survival of the three cohorts after allogeneic stem cell transplantation the difference did not reach level of statistically significance. Visualized in Kaplan-Meier curves allografting appears to be some kind of ‘equalizer’ between the three cohorts. More patients with AML were treated with induction chemotherapy (19 vs. 10 vs. 8%) while 33% of patients with IB2 were treated with best supportive care only compared to 8% with AML (*p* < 0.001). When looking at median overall survival of these patients, there was no difference between the three groups in patients that did undergo induction chemotherapy (13 vs. 16 months) while IB2 patients treated with BSC only did survive longer, nine months compared to two months in patients with AML respectively (*p* < 0.001). These results again underline an existing difference in the disease biology and progression speed of myelodysplastic syndromes with increased blasts compared to patients with AML-MR. Despite the fact that we know about the biologic continuum between MDS and AML, our data could once again show that being biologically similar is not the same as being identical or even comparable. The recently published results of the VERONA-trial emphasized this as well as the trial had failed its primary endpoint of prolonging overall survival in patients with higher-risk MDS treated with hypomethylating agents in combination with venetoclax compared to patients treated with HMA only [17]. Median overall survival difference between the two groups was one month while the VIALE-A trial with the same treatment schemes in older AML patients who were ineligible for intensive treatment could show an overall survival benefit of more than five months [18]. For treatment with hypomethylating agents as well as low-dose chemotherapy we saw a significantly longer median overall survival in MDS patients compared to patients with AML-MR (20 vs. 9 and 11 vs. 1 months), too. These data are in line with multiple previously published trials treating either AML or MDS patients with hypomethylating agents [19–21] and do once again underscore their distinct clinical behavior. The traditional blast cutoff of 20% has been criticized multiple times as arbitrary as patients may become ineligible for either AML or MDS trials [15]. Rigid classification boundaries can distort clinical trial design and limit generalizability of results. From this perspective, a merged or continuum-based classification could improve biological fidelity and facilitate trial inclusion. However, our data showed that even AML-MR and MDS do not only differ in patient demographics and clinical course but also in treatment goals, treatment choice, and overall survival. Patients with MDS are typically older, more comorbid, and often managed with palliative intent, prioritizing quality of life over aggressive disease eradication. In this context, treatment paradigms must focus on more effective but tolerable strategies which we do have available as shown in our median overall survival times in patients with MDS IB2 treated with BSC, low-dose chemotherapy or hypomethylating agents (9, 11 and 20 months). By contrast, using ‘typical’ AML treatment like induction chemotherapy in patients with increased blasts reduced median OS by four months in case of IB2 herewith again underlining the considerable toxicity and loss of benefit for this group of treatment. To our mind, a unified classification therefore risks promoting the inappropriate application of AML-like therapeutic approaches to patients with MDS, raising concerns about overtreatment and treatment-related harm. The indiscriminate extension of intensive therapies may result in a mismatch between treatment burden and clinical benefit, particularly in frail or elderly patients. In addition, merging MDS and AML may paradoxically impair, rather than improve, the interpretability of clinical research as combining biologically and clinically heterogeneous patient populations into a unified study cohort risks diluting treatment effects and obscuring clinically meaningful differences. Thus, while the current separation may introduce artificial distinctions, it also preserves a degree of clinical clarity that remains essential for trial design, regulatory evaluation, and therapeutic decision-making. Our analyses of almost 1000 MDS IB and AML-MR patients diagnosed within approximately the last 40 years has limitations. Looking at the distribution of patients within our cohort, a relevant number of 85% of patients were diagnosed before the year 2020 herewith leading to a time bias. Since genetic analyses have evolved over the last 20 years and molecular testing has become more frequent, there is a huge lack of data making important gain of information like ELN classification as well as risk assessment and prognostication using IPSS-R and IPSS-M of the whole cohort impossible. As our analyses are retrospective and documentation of patients has not always been as extensive and disposable as today, we were not able to give evidence about all end points of interest such as remission or relapse rates as well as treatment-related mortality.

## Conclusion

Taken together, the ongoing debate reflects a fundamental tension between biological accuracy and clinical utility. The significantly longer time to treatment initiation together with the longer overall and post-treatment survival indicates important biological differences between MDS-IB2 and AML-MR thereby calling into question a shared classification. A certain degree of categorical distinction may remain necessary to ensure appropriate treatment selection and maintain the integrity of clinical research.

## Data Availability

The datasets used and analyzed during the current study are available from the corresponding author on reasonable request. The data are not publicly available due to ethical restrictions.
